# Humanization and expression of IgG and IgM antibodies in plants as potential diagnostic reagents for Valley Fever

**DOI:** 10.3389/fpls.2022.925008

**Published:** 2022-09-02

**Authors:** Collin Jugler, Francisca J. Grill, Lukas Eidenberger, Timothy L. Karr, Thomas E. Grys, Herta Steinkellner, Douglas F. Lake, Qiang Chen

**Affiliations:** ^1^The Biodesign Institute, Arizona State University, Tempe, AZ, United States; ^2^School of Life Sciences, Arizona State University, Tempe, AZ, United States; ^3^Department of Applied Genetics and Cell Biology, University of Natural Resources and Life Sciences, Vienna, Austria; ^4^Laboratory Medicine and Pathology, Mayo Clinic, Phoenix, AZ, United States

**Keywords:** coccidioidomycosis, Valley Fever (VF), diagnostics, plant-made diagnostics, IgG, IgM, humanization

## Abstract

Monoclonal antibodies (mAbs) are important proteins used in many life science applications, from diagnostics to therapeutics. High demand for mAbs for different applications urges the development of rapid and reliable recombinant production platforms. Plants provide a quick and inexpensive system for producing recombinant mAbs. Moreover, when paired with an established platform for mAb discovery, plants can easily be tailored to produce mAbs of different isotypes against the same target. Here, we demonstrate that a hybridoma-generated mouse mAb against chitinase 1 (CTS1), an antigen from *Coccidioides* spp., can be biologically engineered for use with serologic diagnostic test kits for coccidioidomycosis (Valley Fever) using plant expression. The original mouse IgG was modified and recombinantly produced in glycoengineered *Nicotiana benthamiana* plants *via* transient expression as IgG and IgM isotypes with human kappa, gamma, and mu constant regions. The two mAb isotypes produced in plants were shown to maintain target antigen recognition to CTS1 using similar reagents as the Food and Drug Administration (FDA)-approved Valley Fever diagnostic kits. As none of the currently approved kits provide antibody dilution controls, humanization of antibodies that bind to CTS1, a major component of the diagnostic antigen preparation, may provide a solution to the lack of consistently reactive antibody controls for Valley Fever diagnosis. Furthermore, our work provides a foundation for reproducible and consistent production of recombinant mAbs engineered to have a specific isotype for use in diagnostic assays.

## Introduction

Antibodies are a leading class of biological molecules utilized in numerous research, diagnostic, and treatment settings. Monoclonal antibodies (mAbs) have a strong research and development interest for therapeutic purposes due to a highly profitable market, predicted to be upward of $300 billion by 2025 (Lu et al., 2020). The market for antibodies as research reagents or diagnostic tools is estimated to reach levels of $6.3 billion by 2027 and $42.56 billion by 2029, respectively (Meticulous Research, 2020, 2022). Many antibodies used for these latter purposes are polyclonal in nature, allowing for more economical production. However, polyclonal antibodies may introduce a large degree of batch-to-batch variation, causing reproducibility issues (Bradbury and Plückthun, 2015; Gray et al., 2020). These issues can be overcome by identifying sequences of mAbs for targets of interest, thereby allowing recombinant production processes to replace mammalian serum collection as an antibody source. Strong market projections, in parallel with the reproducibility crisis, urges innovation in both identifying new mAbs, as well as improving production methods for sustainable, reliable sources for reagents.

Generating mAbs to targets of interest can be accomplished in several ways. Isolation of human B cells, followed by antigen-specific sorting and single-cell variable region sequencing to identify high affinity and specific antibodies is a high throughput method employed when convalescent individuals are available (Gilchuk et al., 2020). However, this method may not be useful for discovering antibodies against antigens that individuals may not been exposed to and thus, do not have B cells that have undergone proper affinity maturation. Also, this method is not cost-effective, requiring cutting-edge technology for successful mAb generation, limiting its accessibility. Phage display is another technology that has generated useful mAbs for therapeutic purposes, although it also has limitations, including libraries not representing all antibodies and potential mispairing of variable heavy and light regions, compared to *in vivo* generation of antibodies (Hammers and Stanley, 2014; Alfaleh et al., 2020). The traditional and widely used method of hybridoma generation following mouse immunization can generate highly pure, full-length mAbs that can be continuously cultured for production (Köhler and Milstein, 1975). However, even hybridoma technology can suffer from instability of the fused cells and there is constant risk of contamination, which can limit sustainable production of a consistent mAb. Furthermore, traditional hybridoma-derived mAbs are of mouse origin, making them unsuitable for human applications. This limitation also pertains to certain *in vitro* diagnostic applications. For example, hybridoma-produced murine mAbs cannot be used as positive controls in commercial assays for diagnosis of antibody responses to pathogens in human patients, as the secondary antibodies (usually anti-human IgG or IgM) cannot recognize the control mAbs of mouse origin.

Recombinant protein expression in plants is an alternative to other expression systems that has gained momentum in recent decades. MAbs against a diverse array of targets have been generated and characterized, with some being produced under current Good Manufacturing Practices (cGMP) as proof-of-principle that plants can be a viable alternative to mammalian-made biopharmaceuticals (Lai et al., 2010; Qiu et al., 2014). There are several advantages of using plants as mAb production hosts. Plants do not require sterile growth conditions or expensive growth media, making them a more cost-effective production method compared to Chinese hamster ovary (CHO) or hybridoma-based methods, while also greatly reducing the risk of contamination of mammalian viruses or other pathogens (Chen et al., 2014; Chen and Davis, 2016). Furthermore, plant host engineering has allowed the production of mAbs in plants with mammalian glycosylation that is more homogenous than those produced in CHO cells, improving the quality and consistency of mAb-based products (Strasser et al., 2008; Chen, 2016). Once the variable gene sequences of a mAb of interest are defined, simple cloning procedures followed by agroinfiltration (Chen and Lai, 2013; Leuzinger et al., 2013) allow the rapid generation of high levels of mAbs in plants. The flexibility of plant-based expression systems has allowed the production of multiple mAbs and mAb variants for a diversity of applications, including engineering the same Fab region to different antibody isotypes and subtypes, and mAbs for diagnostic purposes (Ma et al., 1995; He et al., 2012; Loos et al., 2014; Kallolimath et al., 2021; Sun et al., 2021).

Serological diagnostic tests are used to help identify many different diseases in patients. The specificity of antibodies generated from the immune response against pathogens can be detected by enzyme immunoassays (EIA) to inform the infection status. Coccidioidomycosis (Valley Fever) is a fungal respiratory disease caused by *Coccidioides* spp. (Van Dyke et al., 2019). The primary clinical diagnostic test for Valley Fever detects the antibody response in patient serum to the fungi (Khan et al., 2019). Specifically, patient IgG and IgM against Valley Fever are detected in an EIA by reactivity to *Coccidioides* spp. antigen coated in individual wells on a plate (Malo et al., 2020). Clinical laboratories performing serology tests using Food and Drug Administration (FDA)-approved kits for diagnosis of Valley Fever are required to run dilution controls when testing patient sera, per Clinical Laboratory Improvement Amendments (CLIA) regulations (Centers for Medicare and Medicaid Services and Department of Health and Human Services, 2011), ensuring that patient samples and controls are diluted in the same manner while performing the assay. Since human IgG and IgM dilution controls are not provided in the diagnostic serology kits, human sera that demonstrated previous IgG and/or IgM reactivity to *Coccidioides* spp. antigen must be identified, stored, and re-used with the reagents in the diagnostic kit every time the test is run to comply with federal requirements. The supply of positive human sera is uncertain, and the inherent variability of human sera may lead to inconsistent diagnostic results. The lack of a consistent control presents a major challenge to all clinical laboratories that perform serologic testing for Valley Fever and provides an opportunity to generate human IgG and IgM reagents that can be used as dilution controls with the serologic test kits.

Here, we present the development of a *Coccidioides* spp. antigen-specific mAb produced by murine hybridomas and its conversion to humanized chimeric IgG and IgM isotypes by using a plant-based transient expression system. The two mAb human isotypes are produced efficiently in plants with homogenous mammalian-type glycosylation. Furthermore, they are found to effectively recognize an important *Coccidioides* spp. antigen using reagents similar to those used clinically in Valley Fever diagnostic kits. This study may provide a solution to a particular “pain-point” in clinical laboratories that run serologic tests for the diagnosis of coccidioidomycosis. This may also set a foundation for using plants to provide various human antibody isotypes as human diagnostic reagents, inexpensively and quickly with consistent quality.

## Materials and methods

### Antigen preparation and mouse immunization

Antigen was prepared as a culture filtrate of the mycelial phase of *Coccidioides* spp. as previously detailed (Mead et al., 2020). Mycelial culture filtrate (MCF) was concentrated using an Amicon centrifugal filter unit (Millipore, Burlington, MA, United States) and the total protein concentration was measured with Pierce^™^ BCA Protein Assay Kit (Thermo Scientific, Waltham, MA, United States) according to manufacturer instructions. MCF was mixed with Magic Mouse adjuvant (Creative Diagnostics, Shirley, New York, United States) at 1:1 volume and used to immunize one BALB/c mouse under an Institutional Animal Care and Use Committee–approved protocol (#19-1,684 T) at Arizona State University (50 μg per dose). Subcutaneous (SQ) immunization was repeated with half the dose of MCF at 3-week intervals. Antibody titers to MCF were monitored by indirect ELISA and when an acceptable level was reached (>1:32,000), a final SQ boost was given, and the mouse was sacrificed 3 days later for hybridoma generation.

### Generation and purification of mAbs

Hybridomas were generated using a standard technique (Köhler and Milstein, 1975). Briefly, the spleen was excised and processed into a single-cell suspension, followed by lysis of erythrocytes with 1X red blood cell lysis buffer (Invitrogen, Waltham, MA, United States). The remaining 4 × 10^7^ splenocytes were fused with 8 × 10^7^ murine myeloma cells (P3X63Ag8.653) using 1 ml of 50% polyethylene glycol solution (Sigma-Aldrich, St. Louis, MO, United States). Fused cells were resuspended in 20% fetal bovine serum complete Dulbecco’s modified Eagle medium with hypoxanthine-aminopterin-thymidine selection supplement (Sigma-Aldrich, St. Louis, MO, United States) and plated in 96-well plates at 5 × 10^4^ splenocytes per well. Plates were incubated for 10 days at 37°C in a 5% CO_2_ incubator. Culture supernatant was tested for antibodies to MCF by indirect ELISA. Cells positive by ELISA were subcloned by limiting dilution and screened using the same method after 10 days. Positive subclones were expanded and supernatant was purified by protein A/G chromatography (Thermo Scientific, Waltham, MA, United States). Purified mAbs were evaluated for specificity by Western blotting as described below. One mAb, 4H2, bound to a known antigen in MCF, chitinase 1 (CTS1), and was pursued for gene rescue analyses.

### ELISA for hybridoma screening

Purified MCF was coated on an ELISA plate at 20 μg/ml. Then, the plate was washed with 1X phosphate-buffered saline + 0.05% Tween-20 (PBST) three times and blocked in 1% bovine serum albumin in phosphate-buffered saline (BSA-PBS). For monitoring mouse antibody titers, mouse serum was diluted 1:1,000 in 1% BSA-PBS, then diluted two-fold up to 1:128,000, and allowed to incubate on the ELISA plate for 1 h. For testing hybridoma cultures, neat supernatant was added directly to the blocked ELISA plate and incubated for 1 h. After primary antibody incubation, plates were washed three times with PBST and then incubated with horseradish peroxidase (HRP)-conjugated goat-anti mouse IgG antibody (1:5,000 dilution, Jackson ImmunoResearch, West Grove, PA, United States) for 1 h. Plates were washed four times with PBST and TMB substrate was added. After color development, the reaction was stopped with 0.16 M sulfuric acid, and absorbance was read at 450 nm.

### Mouse immunoglobulin gene rescue

Cell lysate of mAb 4H2 was prepared using the Cells-to-cDNA^™^ II Kit (Invitrogen, Waltham, MA, United States). The cell lysate was subsequently used to synthesize cDNA using a reverse transcription reaction with reagents included in the kit, per the manufacturer’s instruction. The resultant cDNA was subject to polymerase chain reaction (PCR) amplification of unknown mouse immunoglobulin light chain (LC) and heavy chain (HC) variable genes using previously published degenerate primers (Wang et al., 2000). Each PCR reaction contained 2 μl cDNA (Wang et al., 2000), 0.5 μM 5′ and 3′ primers, 25 μl Phusion Flash High-Fidelity PCR Master Mix (Thermo Scientific, Waltham, MA, United States), and ultra-pure water to bring the reaction volume to 50 μl. Cycling conditions were as follows: initial denaturation at 98°C for 10 s followed by 30 cycles of a three-step program (98°C, 1 s; 55°C, 5 s; 72°C, 6 s), and a final extension at 72°C for 1 min. Amplification was confirmed by running 8 μl of the PCR reaction on a 1% agarose gel. The remaining 42 μl of the PCR product were subject to DNA cleanup using the QIAquick^®^ PCR Purification Kit (Qiagen, Hilden, Germany) and the approximate concentration of DNA was determined using a NanoDrop spectrophotometer (Thermo Scientific, Waltham, MA, United States). Cleaned DNA from each PCR reaction was cloned into pcDNA^™^3.1 V5/HisA vector (Thermo Scientific, Waltham, MA, United States) using the Cold Fusion Cloning Kit (System Biosciences, Palo Alto, CA, United States). DNA from resultant clones was extracted using the QIAprep®Spin Miniprep Kit (Qiagen, Hilden, Germany) and sent for Sanger sequencing.

### Human IgG and IgM chimera construction

Primers were designed to add a plant-specific secretion signal peptide to murine variable regions of the LC and HC clones. The resulting PCR products were then digested and ligated onto a human kappa chain backbone, along with a human IgG_1_ (Hurtado et al., 2020) and a human mu chain backbone (Loos et al., 2014), respectively (Supplementary Figure S1), before being cloned into a plant expression vector (a bean yellow dwarf virus-based geminiviral vector pBYR11eK2Md) as described (Jugler et al., 2021). While constant regions of LC and HC were codon-adapted for plant expression, non-plant-adapted codons were maintained in the murine variable regions of the antibody. The sequence of variable and constant regions of IgG and IgM were confirmed at least once. Positive clones containing the human chimeric IgG and IgM constructs were transformed in *Agrobacterium tumefaciens* and verified by PCR.

### Agroinfiltration and temporal expression in *Nicotiana benthamiana*

Transgenes were introduced into glycoengineered ∆XTFT *N. benthamiana* (Strasser et al., 2008) leaves by agroinfiltration as previously described (Leuzinger et al., 2013; Esqueda et al., 2021), and leaves expressing the recombinant IgG or IgM were collected for expression analysis. Leaves collected in the range of 2–9 days post agroinfiltration (dpi) were homogenized in extraction buffer [1X PBS containing 1 mM ethylenediaminetetraacetic acid (EDTA), 2 mM phenylmethylsulfonyl fluoride (PMSF), and 10 mg/ml sodium L-ascorbate] at a 1:1.5 ratio of fresh leaf weight (FLW) to buffer volume. An ELISA plate that had been coated overnight at 4°C with a goat anti-human kappa chain antibody (Southern Biotech, Birmingham, AL, United States) at 2 μg/ml, was washed four times with PBST, followed by blocking with 5% dry milk dissolved in PBST (DM-PBST). Dilutions in DM-PBST of homogenized leaf samples and an isotype antibody control of known concentration were added to the plate and incubated for 1 h at 37°C. After washing four times, a goat anti-human IgG-HRP antibody (1:4,000 dilution, Southern Biotech, Birmingham, AL, United States) or a goat anti-human IgM mu chain-HRP (1:5,000 dilution, Abcam, Cambridge, United Kingdom) was diluted in 5% DM-PBST and added to the plate for 1 h at 37°C. After an additional four washes, TMB substrate was incubated, and reaction was stopped by the addition of 1 M sulfuric acid. Absorbance was read at 450 nm.

### Plant-made mAb purification and SDS-PAGE analysis

*Nicotiana benthamiana* leaves expressing either the humanized 4H2 IgG or IgM were harvested on 7 dpi (4H2 IgG) or 3 dpi (4H2 IgM) and homogenized in the same extraction buffer described above for temporal expression analysis. The protein extract containing IgG or IgM was processed as described (Jugler et al., 2020, 2021), followed by Protein A (Cytiva, Marlborough, MA, United States) or human anti-mu chain (Sigma-Aldrich, St. Louis, MO, United States) affinity chromatography according to the instructions of the manufacturer. Eluted mAbs were further concentrated and buffer exchanged into 1X PBS, pH 7.4 by ultrafiltration in an Amicon centrifugal filter unit (Millipore, Burlington, MA, United States). Purified mAbs were then subjected to SDS-PAGE and Coomassie Blue R-250 staining under both reducing and non-reducing conditions on a Mini-Protean TGX 4%–20% gel (Bio-Rad, Hercules, CA, United States).

### Western blotting

Western blots were performed as described previously (Jugler et al., 2021). In brief, to validate the plant-made mAbs identity, plant-made 4H2 IgG (P-4H2 IgG) or plant-made 4H2 IgM (P-4H2 IgM), along with the IgG (Lai et al., 2010) or IgM (Bio-Rad, Hercules, CA, United States) isotype controls, were subjected to SDS-PAGE under reducing and non-reducing conditions and transferred onto polyvinylidene difluoride (PVDF, Bio-Rad, Hercules, CA, United States) membranes. Membranes were blocked with 5% DM-PBST for 1 h, followed by washing with PBST. Goat anti-human kappa-HRP (1:5,000 dilution, Southern Biotech, Birmingham, AL, United States), goat anti-human IgG-HRP (1:10,000, Southern Biotech, Birmingham, AL, United States) or goat anti-human IgM mu chain-HRP (1:5,000 dilution, Abcam, Cambridge, United Kingdom) were diluted in 5% DM-PBST and incubated for 1 h. After washing with PBST, membranes were developed using SuperSignal® West Pico Chemiluminescent Substrate (Thermo Scientific, Waltham, MA, United States) according to the manufacturer’s instructions and images were taken with an ImageQuant instrument.

For validation of the plant-made mAbs’ target recognition, recombinant CTS1 [sequence confirmed previously (Mitchell et al., 2020)] and *Coccidioides* MCF were separated under reducing conditions on 12% polyacrylamide gels and transferred to PVDF membrane. Following blocking in 1% BSA-PBST, either the parental murine 4H2 IgG (M-4H2 IgG), P-4H2 IgG, or P-4H2 IgM were diluted to 500 ng/ml in 1% BSA-PBS and incubated for 1 h. The membranes were then probed with the appropriate secondary antibody (1:10,000 dilution, goat-anti-mouse IgG Fc-specific-HRP, Jackson ImmunoResearch, West Grove, PA, United States; 1:10,000 dilution, goat-anti-human IgG Fc-specific-HRP, Sigma-Aldrich, St. Louis, MO, United States; 1:10,000 dilution, goat-anti-human IgM Mu-HRP), followed by a 1-h incubation. After washing with PBST, a KPL TrueBlue Peroxidase substrate (SeraCare, Milford, MA, United States) was added, followed by image capture.

### Antigen binding ELISA by chimeric IgG and IgM

To evaluate P-4H2 IgG and P-4H2 IgM antigen binding, recombinant CTS1 was coated on an ELISA plate at 2 μg/ml overnight at 4°C. Then, the plate was washed with 1X PBST three times and blocked in 1% BSA-PBS. MAb isotypes were tested at 1 μg/ml and diluted two-fold in 1% BSA-PBS. After 1 h of incubation, plates were washed three times with PBST and then incubated with HRP-conjugated anti-human secondary antibody (1:20,000 dilution, goat-anti-human IgG Fc-specific-HRP, Sigma-Aldrich, St. Louis, MO, United States; 1:5,000 dilution, goat-anti-human IgM Mu-HRP). Hybridoma-produced parent mAb was used as a positive control, with an HRP-conjugated anti-mouse secondary antibody (1:20,000 dilution, goat-anti-mouse IgG Fc-specific-HRP, Jackson ImmunoResearch, West Grove, PA, United States). Plates were washed four times with PBST and TMB substrate was added. The reaction was stopped with 0.16 M sulfuric acid and absorbance was read at 450 nm.

### N-glycan analysis

The N-glycosylation profiles of the purified Abs were determined by mass spectrometry (MS) as described previously (Sun et al., 2021). Briefly, respective heavy chains were excised from an SDS-PAGE, trypsin digested, and analyzed with an LC-ESI-MS system (Orbitrap Exploris 480, Thermo Scientific, Waltham, MA, United States). The possible glycopeptides were identified as sets of peaks consisting of the peptide moiety and the attached N-glycan varying in the number of HexNAc units, hexose, deoxyhexose, and pentose residues. Manual glycopeptide searches were made using FreeStyle 1.8 (Thermo Scientific, Waltham, MA, United States) and deconvolution was done using the Extract function. The peak heights roughly reflect the molar ratios of the glycoforms. Nomenclature according to Consortium for Functional Glycomics^1^ was used.

### Peptide mapping by mass spectrometry

The target bands between 37 and 50 kDa in the P-4H2 IgM sample were excised from an SDS-PAGE, destained, reduced by the addition of dithiothreitol, and alkylated using iodoacetamide. Trypsin was added and samples incubated at 37°C overnight with shaking. Supernatants were removed from the gel pieces, dried in a SpeedVac and reconstituted in 30 microliters of 0.1% formic acid, and loaded into receiver vials. Liquid chromatography-electrospray ionization tandem mass spectrometry (LC-ESI-MS/MS) analysis was performed according to a protocol described previously (Pabst et al., 2012). Mass spectra were collected and raw files were searched against the UniProt^2^ *Homo sapiens* database (Hsap_UP000005640.fasta) using Proteome Discover 2.5 (Thermo Scientific, Waltham, MA, United States). The MS files were also analyzed using PEAKS (Bioinformatics Solutions Inc., Ontario, Canada), which is suitable for performing MS/MS ion searches. Human IgM heavy chain (HC; NCBI No:AAS01769.1) was used as a reference. A *Nicotiana benthamiana* genome database was used as a control (Schiavinato et al., 2019).

## Results

### Engineering and expression of *Coccidioides*-specific human of IgG and IgM chimeras in *Nicotiana benthamiana*

**Figure 1:**
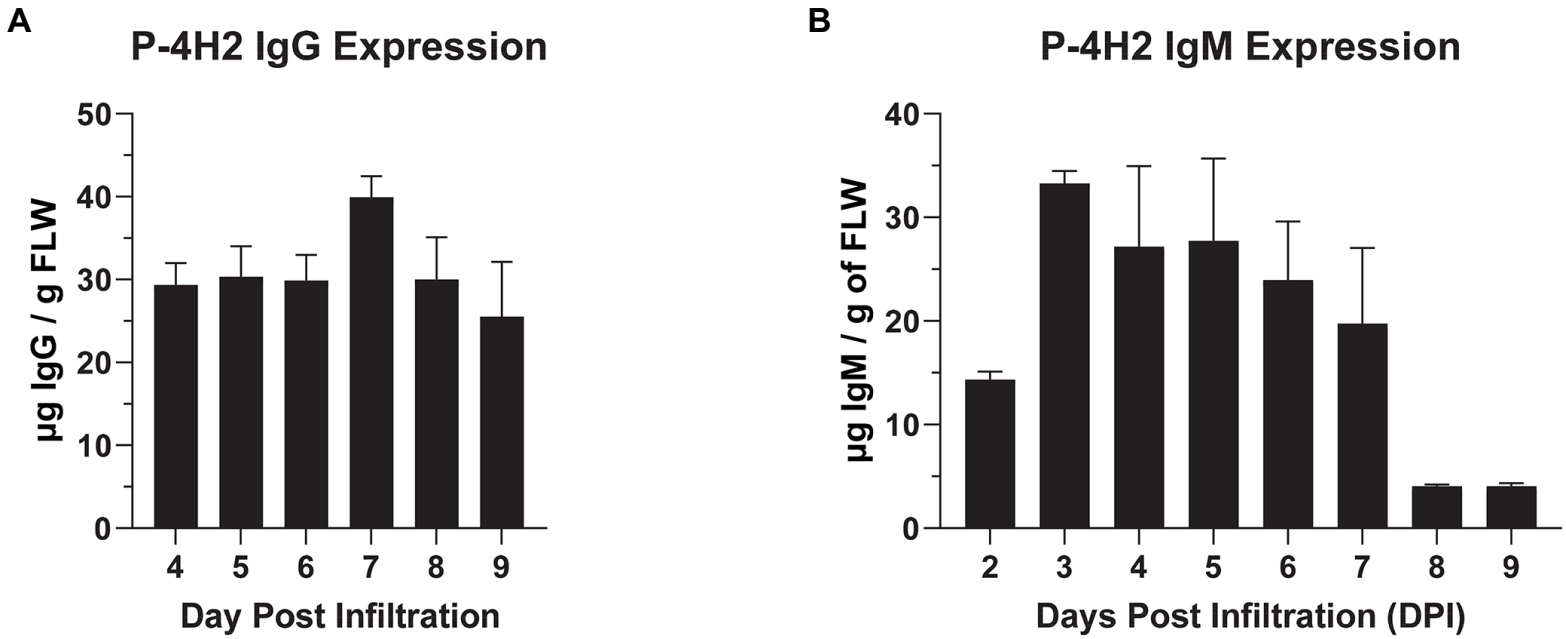
Temporal expression of 4H2 Isotypes in *Nicotiana benthamiana* leaves. ΔXTFT *N. benthamiana* leaves were infiltrated with P-4H2 IgG or P-4H2 IgM gene constructs and leaf proteins were extracted 2–9 days post infiltration (PDI). The expression of P-4H2 IgG **(A)** and P-4H2 IgM **(B)** human chimeras was analyzed by a sandwich ELISA that detects only fully assembled IgG or IgM (containing both human kappa light chain and either human gamma or human mu heavy chain) from total soluble plant protein extracts. Mean ± SEM from at least two independent experiments is plotted.

A mAb of interest, 4H2, was identified by immunizing mice with MCF from *Coccidioides* spp., followed by hybridoma generation and gene rescue. The resulting murine variable heavy region was genetically fused onto both human IgG_1_ and IgM heavy constant chains, in parallel with the variable light region being grafted onto a human kappa constant chain (Supplementary Figure S1). Gene constructs for both humanized IgG and IgM were introduced into leaves of ∆XTFT *N. benthamiana* a glycosylation mutant lacking plant-specific N-glycosylation(Strasser et al., 2008). Recombinant expression of the 4H2 IgG and IgM chimeras was monitored over time by an anti-human IgG or IgM ELISA. Peak expression of P-4H2 IgG was observed at day 7 after agroinfiltration, reaching 39.95 ± 2.5 μg/g FLW (Figure 1A). P-4H2 IgM reached peak expression of 33.3 ± 1.19 μg/g FLW at day 3 after introduction of the transgenes (Figure 1B).

### Recombinant chimeric IgG and IgM purification and characterization

**Figure 2:**
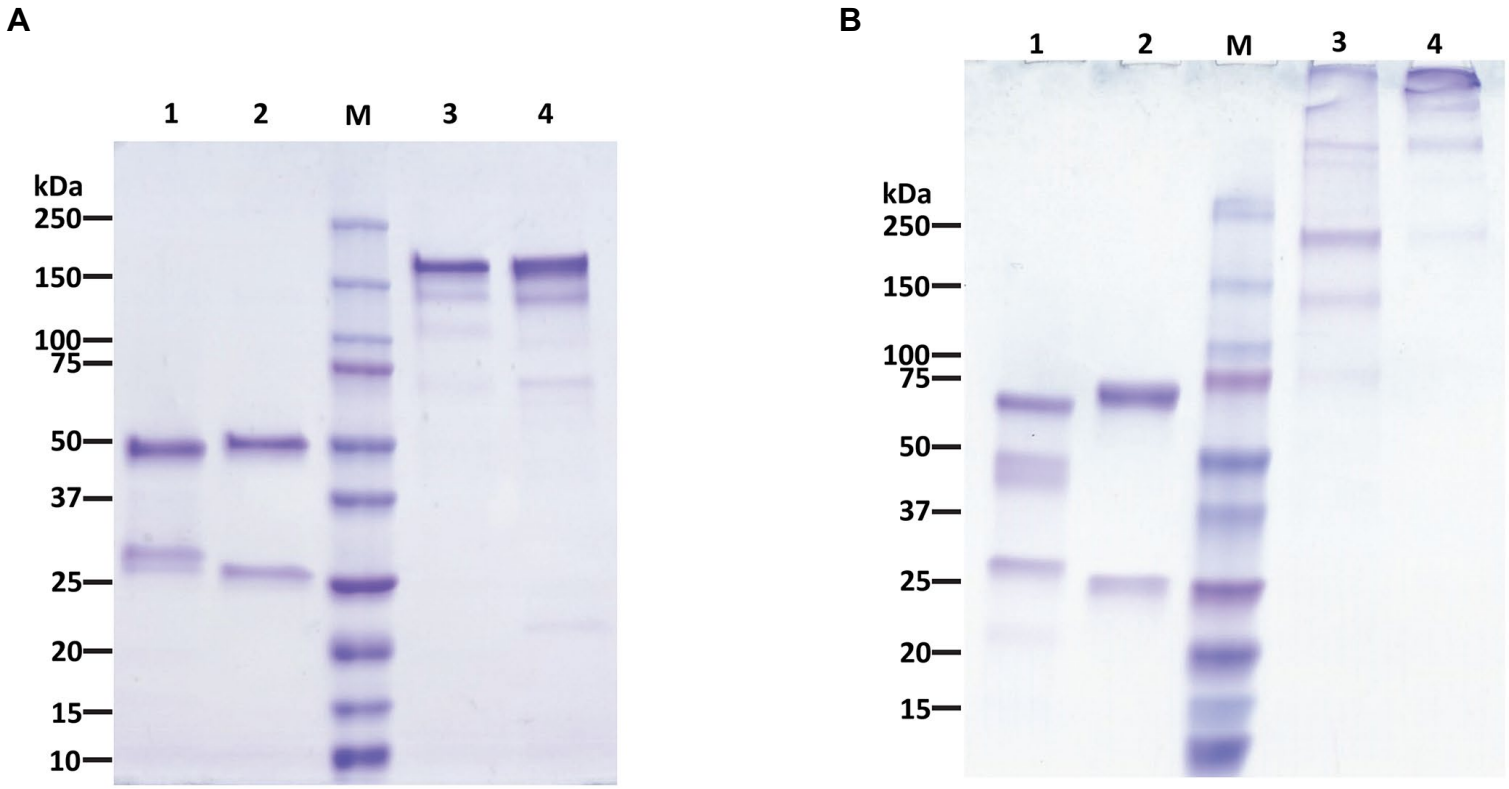
Purification of P-4H2 IgG and P-4GH2 IgM from *Nicotiana benthamiana* leaves. Affinity-purified, humanized P-4H2 IgG **(A)** and P-4H2 IgM **(B)** were separated by SDS-PAGE on 4%–20% polyacrylamide gels under reducing (lanes 1 and 2) and non-reducing (lanes 3 and 4) conditions. One representative of multiple experiments is shown. Lanes 1 and 3, plant-made P-4H2 IgG **(A)** or P-4H2 IgM **(B)**. Lanes 2 and 4, human IgG **(A)** or IgM **(B)** isotype control. M, molecular weight marker. 2.5 μg of protein was loaded per lane.

**Figure 3:**
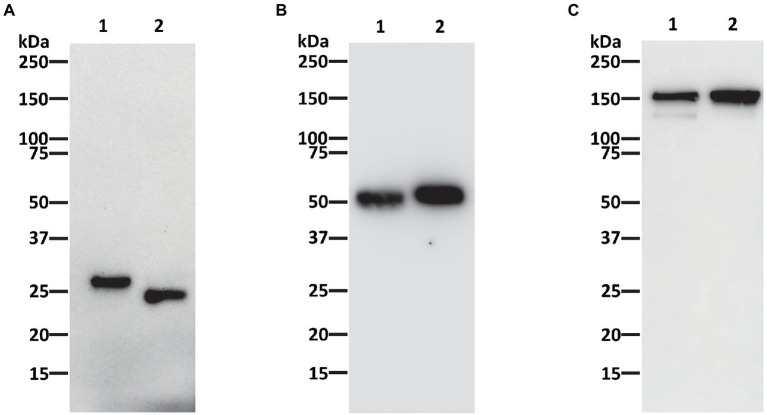
Western blot analysis of 4H2 IgG purified from *Nicotiana benthamiana* leaves. Purified recombinant P-4H2 IgG was separated under reducing **(A,B)** or non-reducing **(C)** conditions by SDS-PAGE and proteins was transferred to PVDF membranes. Proteins were then detected with antibodies specific for human kappa chain **(A,C)** or human gamma chain **(B)** to verify protein identity and assembly. Lane 1, Humanized P-4H2 IgG (1 μg per lane in **A**, 0.5 μg per lane in **B,C**), Lane 2, Human kappa IgG isotype reference standard (1 μg per lane in **A**, 0.5 μg per lane in **B,C**).

**Figure 4:**
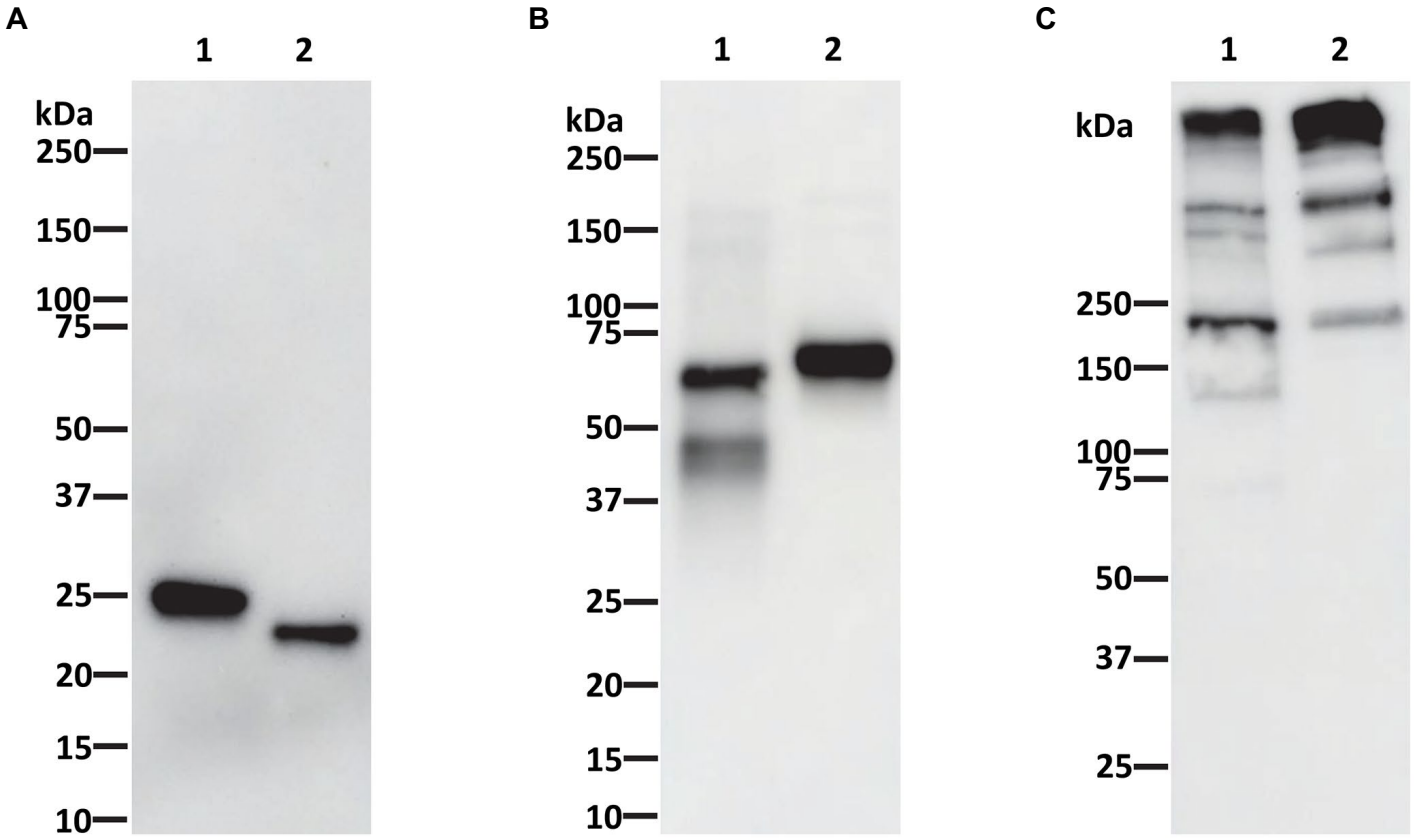
Western blot analysis of purified P-4H2 IgM. Recombinant 4H2 IgM purified from *Nicotiana benthamiana* leaves was separated under reducing **(A,B)** or non-reducing **(C)** conditions by SDS-PAGE and proteins were then transferred to PVDF membranes. The membranes were incubated with antibodies against human kappa chain **(A,C)** or human mu chain **(B)** to verify protein identity and IgM assembly. Lane 1, Humanized P-4H2 IgM (1 μg per lane in **A**, 0.5 μg per lane in **B,C**), Lane 2, Human kappa IgM isotype control (1 μg per lane in **A**, 0.5 μg per lane in **B,C**).

P-4H2 IgG was purified to a high degree of homogeneity by Protein A affinity chromatography, comparable to an isotype control IgG mAb (Figure 2A). The recombinant P-4H2 IgM was purified by affinity chromatography with a goat anti-human mu chain agarose resin (Figure 2B). Next to the expected HC and LC of P-4H2 IgM (~62 and ~26 kDa, respectively), additional bands were observed at molecular mass slightly below 50 kDa (Figure 2B, Lane 1). Western blot analyses were performed to verify the identity and proper assembly of P-4H2 IgG and P-4H2 IgM. The results confirm that the bands observed with SDS-PAGE analysis of the IgG isotype indeed correspond to both human kappa chain at ~26 kDa (Figure 3A), as well as human gamma chain at ~50 kDa (Figure 3B). Likewise, protein bands representing human kappa chain (~ 26 kDa, Figure 4A), as well as the human mu chain (~62 kDa, Figure 4B), were also confirmed for P-4H2 IgM. Moreover, the unexpected bands in the P-4H2 IgM sample reacted with the anti-human mu chain antibody (Figure 4B, Lane 1), suggesting mu HC degradation. Further analysis with mass spectrometry confirmed that the bands are indeed derived from the mu heavy chain (Supplementary Figure S2). Specifically, human IgM mu HC was identified by peptide mapping with a sequence coverage of 89%. Nevertheless, the results do not point to a specific cleavage product, but rather to an unspecific degradation or an inherent instability of the IgM HC.

As expected, P-4H2 IgG under non-reducing conditions is composed of two HC and two LC, for assembled IgG size of ~150 kDa (Figure 3C). Similar banding patterns were observed for P-4H2 IgM and the human kappa IgM isotype control, with the expected monomeric size of ~170 kDa and bands >250 kDa which may represent the larger molecular forms such as penta- or hexamers (Figure 4C).

As glycosylation is an import quality parameter of mAbs, this post-translational modification was determined in detail by MS methods. N-glycan analysis of ΔXTFT produced 4H2 IgG exhibited a largely homogeneous glycosylation profile, with complex N-glycans lacking core xylose and fucose (~80%; GnGn, GnM structures, Table 1). Glycosylation of IgM is more complex as it carries five glycosylation sites (GS1–GS5) for specific glycosylation (Loos et al., 2014). ΔXTFT derived P-4H2 IgM carried mainly GnGn/GnM structures at GS1-3 (between 70% and 80% depending on the GS), while GS4 is decorated with oligomannosidic structures (GS5, located at the tailpiece, is not present in this construct; Table 1). Collectively, compared to mammalian cell-produced mAbs that usually carry more than 10 heterogeneous glycoforms (Yang et al., 2018), plant-expressed 4H2 isotypes exhibit a largely homogenous glycosylation profile with batch-to-batch consistency.

**Table 1:** N-linked glycosylation of 4H2 mAb isotypes.

	4H2 IgG (%)	4H2 IgM (%)			
	Fc	GS1	GS2	GS3	GS4
GnGn	68	17	63	69	
GnGnF			6	5	
GnM	14	62	14	14	
MM	10				
Man	8				90
Diverse		23	19	12	10

N-glycosylation profile was determined by liquid chromatography–electrospray ionization–mass spectrometry (LS-ESI-MS). Numbers represent the presence of various 4H2 mAb glycoforms in percentage. Glycans were annotated according to the ProGlycAn nomenclature (www.proglycan.com). Fc, Fragment, crystallizable region of the antibody; GS1-4, glycosites 1 to 4 of IgM.

**Figure 5:**
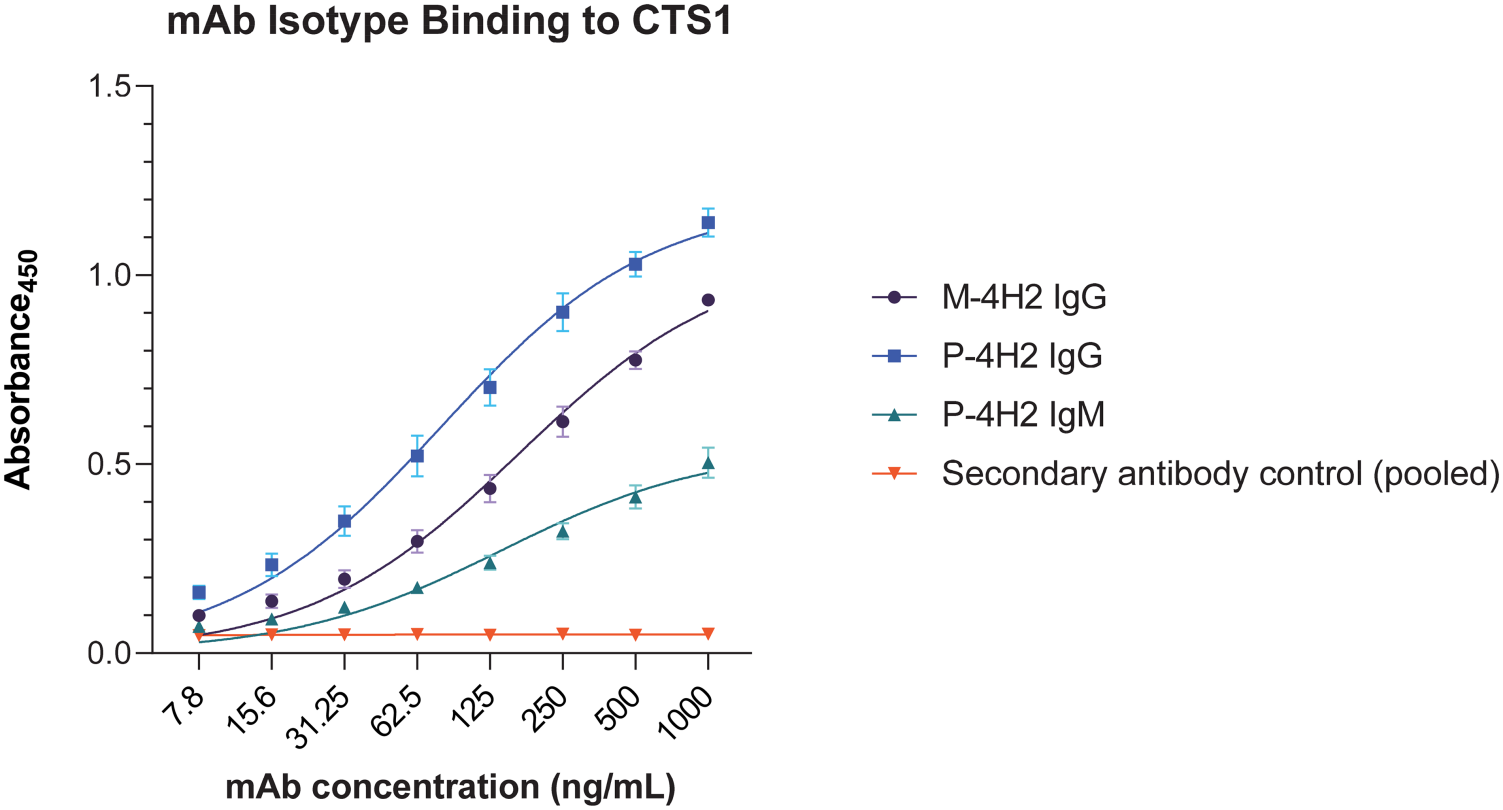
Plant-Made 4H2 Isotypes Specifically Bind to Target Antigen. Serial dilutions of P-4H2 IgG and P-4H2 IgM isotypes were incubated with CTS1 immobilized on ELISA plates. An HRP-conjugated anti-human IgG or anti-human IgM secondary antibody was used to detect the specific interaction between the plant-made IgG or IgM isotype (P-4H2 IgG, P-4H2 IgM) with CTS1, respectively. The parental mouse IgG (M-4H2 IgG) was used as a positive control, which was detected with an anti-mouse IgG secondary antibody conjugated to HRP. Mean ± SEM is plotted from three independent experiments.

**Figure 6:**
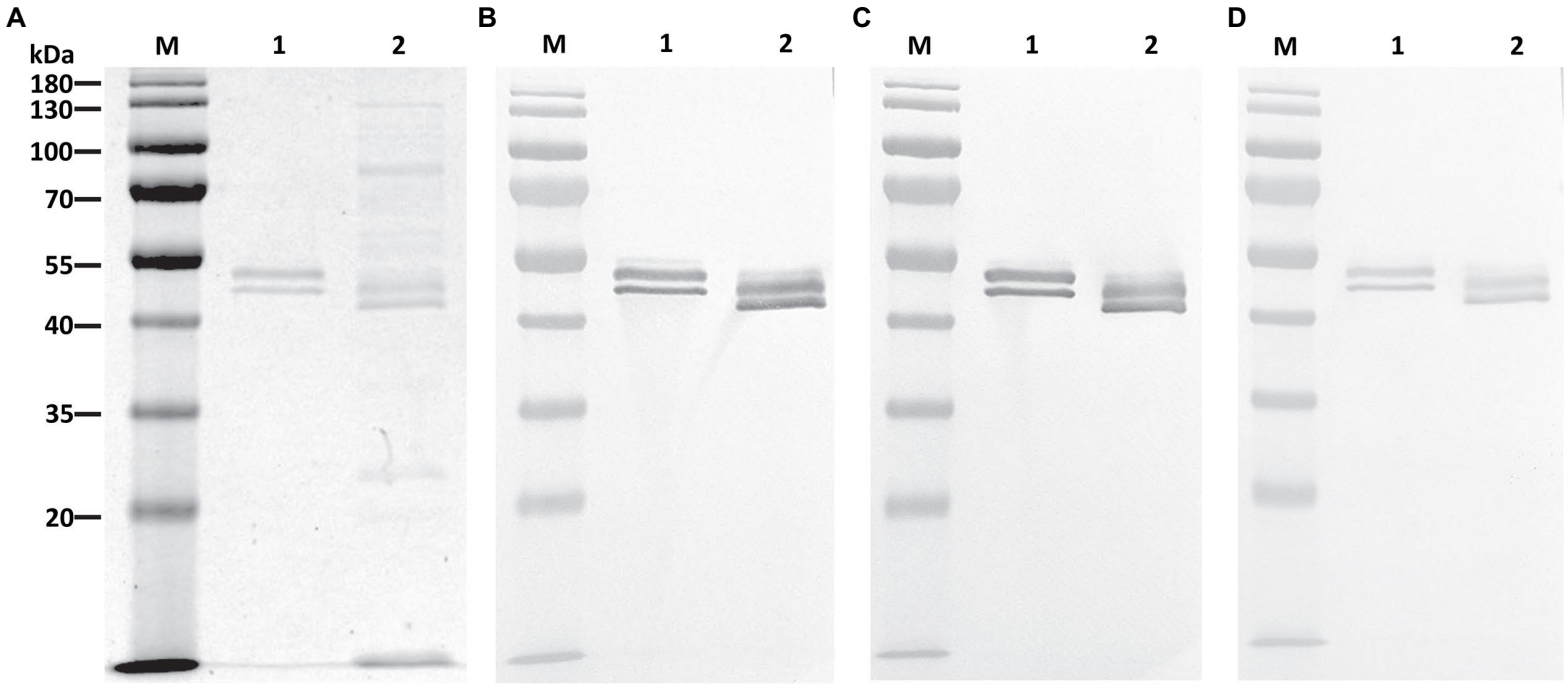
Plant-Made 4H2 Isotypes Bind to Target Antigen on Western Blot. Recombinant CTS1 and *Coccidioides posadasii* mycelial culture supernatant were separated by SDS-PAGE (12%) under reducing conditions and either stained with Coomassie Blue **(A)** or transferred to PVDF membrane. Membranes were then probed with the parental M-4H2 IgG **(B)**, the humanized, P-4H2 IgG **(C)**, or the humanized, P-4H2 IgM **(D)**, followed by the appropriate secondary HRP-conjugated antibody and development with a colorimetric substrate. Lane 1, Recombinant CTS1. Lane 2, *Coccidioides posadasii* mycelial culture supernatant. M, molecular weight marker.

Since chitinase 1 (CTS1) is an important immunogenic component of MCF and commercial antigen preparations used clinically in Valley Fever diagnostic kits (Pishko et al., 1995; Mitchell et al., 2020; Grill et al., 2021), we anticipated that the plant produced mAb bound to *Coccidioides* CTS1. We therefore tested P-4H2 IgG and IgM for the ability to bind to recombinant CTS1 as well as CTS1 in MCF. As determined by indirect ELISA, both recombinant isotypes bound to recombinant CTS1 in a dose-dependent manner (Figure 5), with nearly identical activity to M-4H2 IgG. Similarly, western blot analysis also indicated specific interaction between the plant-made IgG and IgM mAbs and both recombinant CTS1 and MCF (Figure 6), further validating the binding activity of the P-4H2 IgG and P-4H2 IgM to the antigen of interest.

## Discussion

In this study, we utilized plants as an alternative recombinant protein expression platform to express two isotypes of a hybridoma-produced mAb against a fungal antigen, CTS1, from *Coccidioides* spp. To our knowledge, this is only the second report of generating an IgM molecule in plants, but a first for our intentional monomeric IgM design (pairing of two HC), as opposed to a pentameric design (Loos et al., 2014; Vasilev et al., 2016). Both the humanized P-4H2 IgG and P-4H2 IgM were efficiently expressed within a week of transgene delivery, with levels of 39.95 ± 2.5 and 33.3 ± 1.19 μg/g FLW, respectively. These expression levels fall within the range of previously reported plant-made mAbs, but recombinant IgG levels vary widely, with observations ranging from 4 to 1,500 μg/g FLW(Diamos et al., 2020; Shanmugaraj et al., 2020), while the previous expression of a pentameric IgM reached levels of approximately 84 μg/g FLW (Loos et al., 2014). The moderate expression levels of the IgG and IgM observed in this study may be primarily attributed to the variable domains. First, the amino acid sequence and the derived structure of the variable domains of this mAb may have contributed to the decreased expression, or more importantly, non-plant codon-optimized sequences were used for the variable domains. For example, 63.6% and 61.3% codons in the current variable domains of HC and LC constructs, respectively, are different from the typical codons used by genes of *N. benthamiana* plants. We also used a version of the geminiviral expression vector that is different than those reported in the literature for high-level antibody expression, such as MagnICON (Giritch et al., 2006) and the improved geminiviral vectors (Diamos et al., 2020). Preliminary results indicate that indeed expression levels of the two antibody isotypes can be increased, however further studies are necessary before drawing a final conclusion.

The sandwich ELISA used to monitor expression level was designed to only recognize recombinant mAbs that contained both a human kappa LC and either a human gamma (for IgG) or human mu (for IgM) HC. The proper assembly of both the IgG and IgM monomers was further validated by reducing and non-reducing western blot. Indeed, the IgG kappa chain was detected at the expected size of ~150 kDa. Furthermore, the kappa and mu chain components of the IgM observed in SDS-PAGE were identified and pairing of two mu HC and two kappa LC to form a monomeric IgM was shown to occur, indicated by the ~170 kDa band under non-reducing conditions both on western blot and SDS-PAGE. We expected no more than a monomeric form of the IgM, due to the intentional absence of the joining (J)-chain gene, yet a similar band pattern can be observed when comparing the plant-made monomeric IgM with the pentameric human IgM control, indicating possible formation of other IgM oligomers without the J-chain, which has been observed in other studies (Cattaneo and Neuberger, 1987; Randall et al., 1992a,b; Niles et al., 1995; Hiramoto et al., 2018).

Both the chimeric P-4H2 IgG and P-4H2 IgM were purified by simple affinity chromatography methods, specific for their respective heavy constant regions. The IgG reached comparable levels of purity to a mammalian cell-produced IgG subjected to the same purification scheme. However, in purified P-4H2 IgM samples, HC-associated degradation products were detected, which were not seen in the IgM control made in mammalian cells. However, peptide mapping analyses by mass spectrometry did not show specific proteolytic products with defined protease cleavage sites, as occasionally observed for IgG antibodies (Puchol Tarazona et al., 2020), indicating unspecific degradation. To reduce P-4H2 IgM HC degradation, one strategy is to co-infiltrate the J chain construct along with those of the LC and HC to form pentameric IgM structures (Loos et al., 2014), which would likely increase the stability of the protein, help protein folding, and may make it less prone to proteolytic degradation during protein extraction and purification. The inclusion of protease inhibitors during mAb extraction and purification may also limit proteolysis. If proteolysis cannot be avoided, additional purification steps can be applied. For example, a second affinity chromatography step specific for human kappa chain can be included to enrich functional species of IgM that have both mu and kappa chains. This would provide a consistent product for the intended use as an antigen-specific human dilution control.

∆XTFT produced 4H2 IgG and 4H2 IgM exhibited a largely homogeneous N-glycosylation profile, with one dominant glycan species, namely GnGn/GnM structures. Reliable batch-to-batch glycan consistency was obtained, a typical feature of antibodies produced in ∆XTFT (Strasser et al., 2008). Compared to mammalian cells with a high degree of glycan diversity that is hard to control (Yang et al., 2018), the approach described here provides an obvious advantage to ensure the quality and consistency of the diagnostic product.

Valley Fever is a fungal disease that requires an accurate and timely diagnosis. However, current FDA-approved diagnostic kits lack a dilution control. Currently, clinical laboratories that run serologic tests to detect antibodies against *Coccidioides* spp. are forced to identify, collect, store and re-use previously IgG and IgM-positive sera for use in subsequent test runs. This can lead to inconsistency in diagnoses due to inherent variability of storing and re-using human sera. In response to this unmet need for a consistent diagnostic control, we developed a mouse mAb specific to a *Coccidioides* spp. antigen used in the clinical diagnostic kits and engineered it into human IgG and IgM chimeras. We confirmed that the human chimeras retain original antigen recognition by indirect ELISA with recombinant CTS1. This binding was validated in parallel by Western blotting using recombinant CTS1, alongside the immunogen, MCF. Taken together, these data provide evidence that the plant-made chimeric IgG and IgM specifically recognize an antigen of interest (CTS1) found in the immunogen, in a manner consistent with the parental murine mAb. Furthermore, the humanization allows these chimeric mAb isotypes to be recognized by anti-human IgG and IgM secondary antibodies in an ELISA, like those used in Valley Fever diagnostic kits. Therefore, these human chimeric mAbs may be useful as positive controls to replace positive human sera currently used to comply with federal law. This may lead to more consistent diagnostic results and a sustainable supply. The next phase of research will involve investigating the utility of these mAbs in the commercial kits with clinical samples and gaining regulatory approval. Overall, our study highlights the opportunity of plant-based, recombinant production of both IgG and IgM isotypes against CTS1 as consistent controls for Valley Fever diagnostic kits. This platform may provide a consistent and reproducible source of low-cost mAbs as diagnostic reagents for other diseases as well.

## Data availability statement

The original contributions presented in the study are included in the article/Supplementary material, further inquiries can be directed to the corresponding author.

## Ethics statement

The animal study was reviewed and approved by Institutional Animal Care and Use Committee at Arizona State University.

## Author contributions

QC and DL conceptualized research. CJ, FG, LE, and TK designed experiments, performed experiments, and analyzed data. CJ, FG, and QC wrote the paper with revision by DL, HS, and TG. All authors contributed to the article and approved the submitted version.

## Conflict of interest

The authors declare that the research was conducted in the absence of any commercial or financial relationships that could be construed as a potential conflict of interest.

## Publisher’s note

All claims expressed in this article are solely those of the authors and do not necessarily represent those of their affiliated organizations, or those of the publisher, the editors and the reviewers. Any product that may be evaluated in this article, or claim that may be made by its manufacturer, is not guaranteed or endorsed by the publisher.

## Footnotes

^1^ http://www.functionalglycomics.org

^2^ www.uniprot.org

## Supplementary material

SUPPLEMENTARY FIGURE S1 Diagram of P-4H2 IgG and P-4H2 IgM constructs. The DNA constructs encoding the heavy and light chain of P-4H2 IgG and P-4H2 IgM and their sizes in base pairs (bp) are illustrated. Corresponding amino acid numbers in light and heavy chain and their approximate molecular weights predicted (based on peptide backbone only) are also indicated. The pairing of the light chain with either the IgG or IgM heavy chains is indicated by arrows, and the predicted molecular weight of assembled IgG and monomeric IgM is illustrated. MW, molecular weight; bp, base pair. The diagram was created in BioRender.com.

SUPPLEMENTARY FIGURE S2 Peptide mapping by mass spectrometry. Tryptic digest of the unexpected bands (between 37 and 50 kDa) in the purified P-4H2 IgM sample was analyzed by LC-ESI-MS/MS. Spectra were searched against a FASTA database of human proteomes (obtained from UniProt) using Proteome Discover 2.5. Human IgM heavy chain (mu) constant domain (accession no. P01871) was identified with 89.58%. Spectra were also analyzed by PEAKS against the full amino acid sequence of P-4H2-IgM mu HC, which is presented in the figure. Sequence highlighted in gray indicates regions with peptides identified in the unexpected bands (between 37 and 50 kDa). Blue bars illustrate the frequency of detection for individual peptides. Amino acids with orange and red highlights indicate carbamidomethylation and oxidation sites, respectively.
