# Supplementary figures and images for: Humanization and expression of IgG and IgM antibodies in plants as potential diagnostic reagents for Valley Fever
Source: Front Plant Sci. 2022 Sep 2;13:925008. doi: 10.3389/fpls.2022.925008 (PMC9478164; doi:10.3389/fpls.2022.925008)

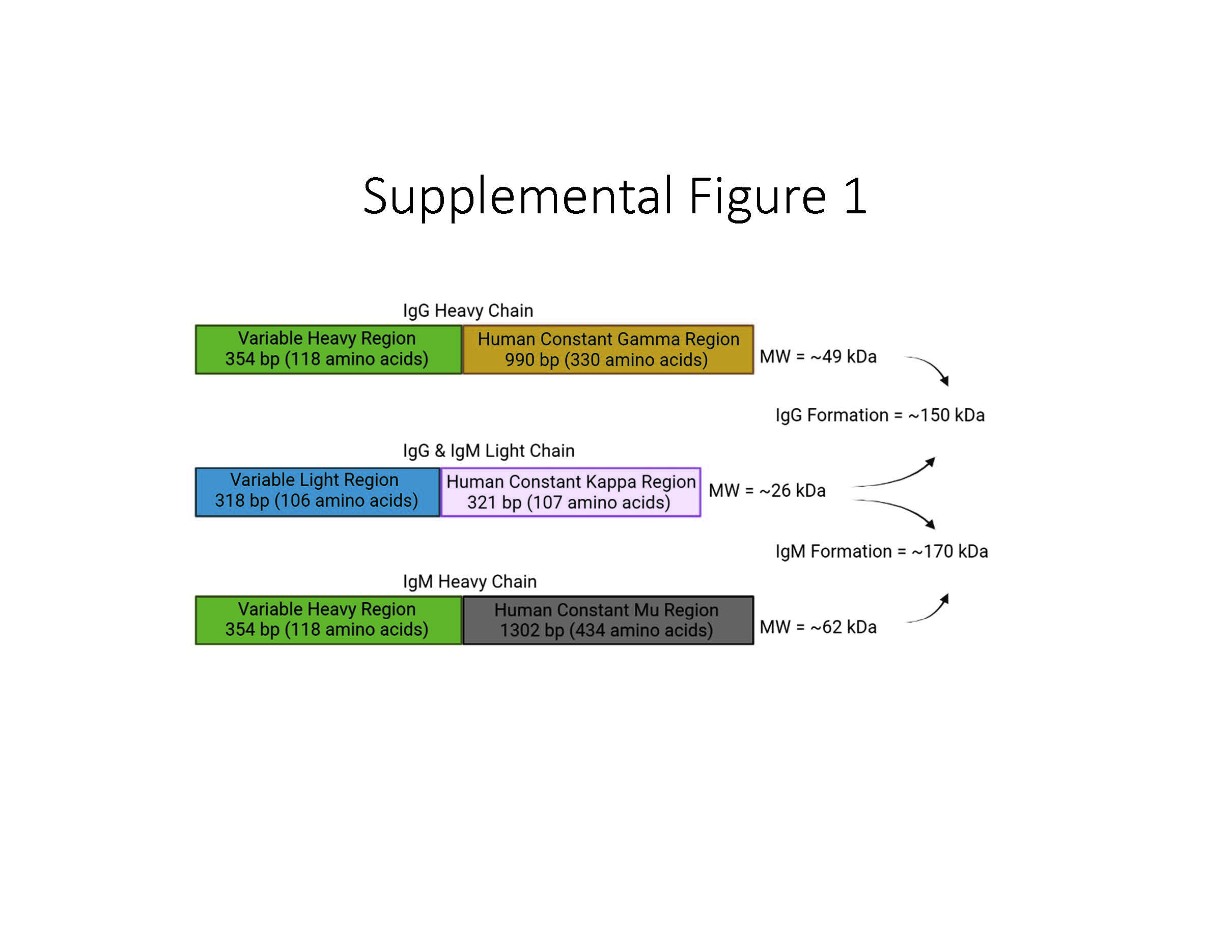

Supplement: SUPPLEMENTARY FIGURE S1 — Diagram of P-4H2 IgG and P-4H2 IgM constructs. The DNA constructs encoding the heavy and light chain of P-4H2 IgG and P-4H2 IgM and their sizes in base pairs (bp) are illustrated. Corresponding amino acid numbers in light and heavy chain and their approximate molecular weights predicted (based on peptide backbone only) are also indicated. The pairing of the light chain with either the IgG or IgM heavy chains is indicated by arrows, and the predicted molecular weight of assembled IgG and monomeric IgM is illustrated. MW, molecular weight; bp, base pair. The diagram was created in BioRender.com. [file Image_1.jpg]

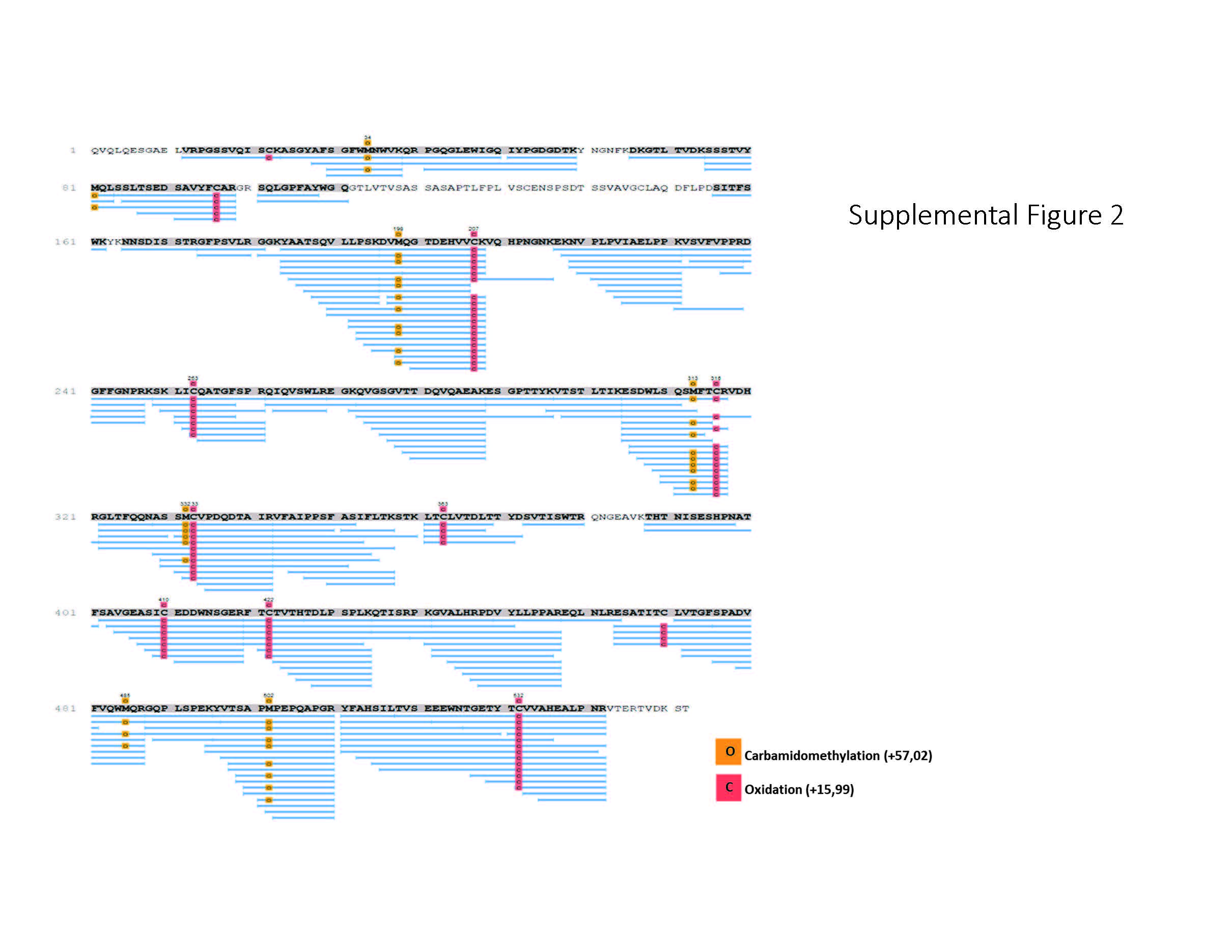

Supplement: SUPPLEMENTARY FIGURE S2 — Peptide mapping by mass spectrometry. Tryptic digest of the unexpected bands (between 37 and 50 kDa) in the purified P-4H2 IgM sample was analyzed by LC-ESI-MS/MS. Spectra were searched against a FASTA database of human proteomes (obtained from UniProt) using Proteome Discover 2.5. Human IgM heavy chain (mu) constant domain (accession no. P01871) was identified with 89.58%. Spectra were also analyzed by PEAKS against the full amino acid sequence of P-4H2-IgM mu HC, which is presented in the figure. Sequence highlighted in gray indicates regions with peptides identified in the unexpected bands (between 37 and 50 kDa). Blue bars illustrate the frequency of detection for individual peptides. Amino acids with orange and red highlights indicate carbamidomethylation and oxidation sites, respectively. [file Image_2.jpg]
